# Structural and optical properties of ligand-free noble metal nanoclusters synthesized by ns-pulsed laser ablation at the argon–water interface

**DOI:** 10.1039/d6na00468g

**Published:** 2026-09-15

**Authors:** Guilherme Conceição Concas, Mariana Gisbert, Tahir, Natan Gonzaga, Francesco Enrichi, Anna Safonova, Michele Cassetta, Sven Reichenberger, Theo Fromme, Alexandre Cuin, Ricardo Aucelio, Tatiana Dillenburg Saint'Pierre, Isabel Cristina dos Santos Carvalho, Wanessa Afonso de Andrade, Rajwali Khan, Zubair Ahmed, Sajid Farooq, Alexandre Mello, Nicola Daldosso, Tommaso Del Rosso

**Affiliations:** a Department of Physics, Pontifical Catholic University of Rio de Janeiro Rua Marquês de São Vicente 225 22451-900 Gávea Rio de Janeiro Brazil tommaso@puc-rio.br; b Department of Engineering for Innovation Medicine, University of Verona Strada le Grazie 15 37134 Verona Italy guilherme.conceicaoconcas@univr.it; c Department of Physics, Federal University of Pelotas Rua dos Ipês - Capão do Leão RS 96050-500 Brazil; d Department of Technical Chemistry I, University of Duisburg-Essen Universitätsstraße 5 45141 Essen Germany; e Departamento de Química, Instituto de Ciências Exatas, Universidade Federal de Juiz de Fora Juiz de Fora MG 36036-900 Brazil; f Department of Chemistry, Pontifical Catholic University of Rio de Janeiro Rua Marquês de São Vicente 225 22451-900 Gávea Rio de Janeiro Brazil; g National Water and Energy Center, United Arab Emirates University Al Ain 15551 United Arab Emirates; h School of Chemical Sciences, Universiti Sains Malaysia Gelugor 11800 Pulau Pinang Malaysia; i College of Mathematical Medicine, Zhejiang Normal University Jinhua 321004 PR China; j Laboratory of Surfaces and Nanostructures (LabSurf), Brazilian Center for Physics Research (CBPF) 22290-180 Rio de Janeiro RJ Brazil

## Abstract

Fully inorganic ligand-free noble metal nanoclusters (NCs) are a challenging system to obtain due to the fact that conventional chemical synthesis introduces surface-bound contaminants. Here, we present a one-step method for synthesizing highly pure, photoluminescent gold (Au) and silver (Ag) NCs by using Pulsed Laser Ablation at the argon–water interface to ensure an entirely CO_2_-free ablation environment. The colloids generated contain a mixture of larger plasmonic nanoparticles and small photoluminescent NCs that have been separated by centrifugation to yield ultrasmall NCs with a mean radius of about 1.8 nm (Au) and 1.4 nm (Ag). Comprehensive analysis by means of TEM, HRTEM, XRD, XPS, surface-enhanced Raman spectroscopy (SERS), and steady-state and time-resolved photoluminescence was performed to assess the absence of carbonaceous contaminants and to give a clear structural, optical, and chemical characterization of the two systems. XPS measurements demonstrated that the surface of the nanostructures exhibited predominantly metallic contributions. However, the silver nanostructures exhibited approximately 20% oxidation, while the gold nanostructures exhibited approximately 4%. Both Au and Ag NCs exhibit similar photoluminescence in both the UV and visible (blue) regions, with Quantum Yields (QY) around 1.2% (Au) and 2.0% (Ag) in the blue region. Time-resolved measurements reveal nanosecond-scale decays with two components: a fast decay of 2–3 ns and a slower decay of 6–9 ns. Notably, the fast component contributes ≈80% of the decay for Au NCs, but only ≈50% for Ag NCs, indicating differences in excited-state relaxation pathways specific to the metal, probably associated with the different degree of oxidation. Surprisingly, UV-Vis absorption spectroscopy reveals a strong absence of the conventional Localized Surface Plasmon Resonance (LSPR) band, showing instead a monotonic, molecular-like interband absorption profile. We hypothesize that this anomalous suppression of the LSPR peaks may be linked to unique features of the PLAL process, such as surface oxidation and a polycrystalline structure, which may artificially trigger quantum-size effects in 3.5 nm particles by confining the electronic response within subcrystalline grains. These fully inorganic NCs remain stable in water, providing a clean platform for understanding the fundamental properties of light behavior in noble metal nanostructures.

## Abstract 2

Fully inorganic photoluminescent Au and Ag nanoclusters produced by CO_2_-free pulsed laser ablation in liquid exhibit anomalous LSPR suppression, and oxidation-tuned PL decay.

## Graphical abstract

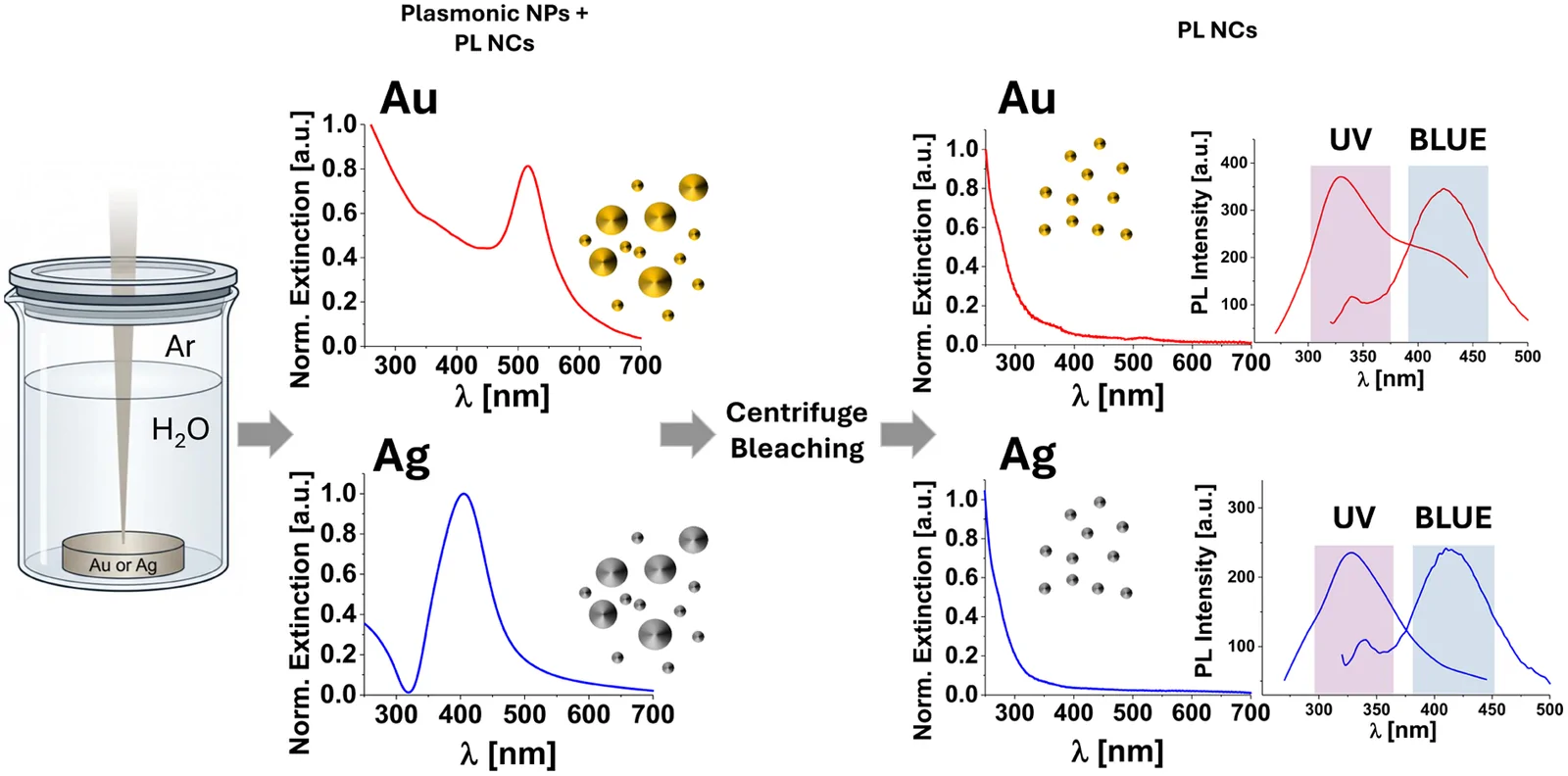

## 1 Introduction

In recent years, laser-assisted fabrication and processing methodologies have fundamentally transformed modern materials science, engineering functional architectures with unprecedented precision across diverse fields ranging from surface science^1,2^ to advanced device manufacturing.^3,4^ Within this context, techniques operating *via* pulsed laser deposition on substrates (PLD) and laser processing and synthesis of colloids in liquids (LSPC) have emerged as two highly complementary and powerful routes.^5–8^ Across both technological landscapes, noble metal nanostructures based on gold and silver remain the most fascinating candidate systems for photonics and optoelectronics. Their widespread appeal stems from a unique dual nature: while larger plasmonic nanoparticles exhibit strong localized surface plasmon resonance (LSPR) ideal for label-free solution concentration analysis and enhanced sensing geometries,^9–11^ ultrasmall metallic nanoclusters undergo extreme quantum confinement, triggering molecule-like optical transitions and efficient photoluminescence.

Noble metal nanoclusters are attracting increasing interest in the research community, not only because of their numerous applications such as in catalytic processes for methanol and ethanol oxidation,^12^ photonics with the study of fundamental optical properties of nanostructures,^13^ chemical sensing of heavy metal pollutants in water,^14,15^ or nanomedicine as luminescent probes,^10,16,17^ but also because they represent a few atoms of light-emitting species, which allow to perform investigations in fundamental physics and chemistry.^18–20^ A great deal of experimental and theoretical work can be found in the literature on the origin of the photoluminescence of metal quantum dots. Considering a few atoms metal nanoclusters, the free-electron Jellium model has a considerable success in modeling the relative optical properties, where the simple scaling relation 
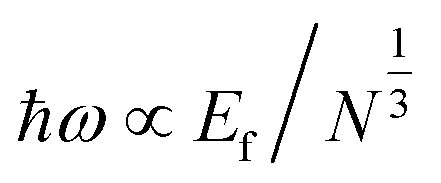
 relates the frequency of the emitted photons to the Fermi energy of the metal and the number of atoms in the clusters.^18^ These multi-electron artificial atoms characterized by quantum confinement can be perfectly engineered by chemical reduction of gold salts in dendrimers, revealing the electronic magic cluster effect, where only magic numbers of atoms allow the stability of the clusters in liquid phase.^21^ On the other hand, the Jellium model fails to model larger clusters, even after the introduction of anharmonic potentials, and cannot predict the effect of organic ligands on the emission properties, such as in the case of the well-known Au_*x*_(SG)_*y*_ quantum dots (SG = glutathione).^22^ In the latter case, as well as in other gold- or silver-based nanoclusters stabilized by organic ligands, the organic species seem to play a fundamental role in mediating the electron–hole recombination through low-energy surface states, allowing the control of both the quantum yield and the emission frequency.^23,24^ The valence state of the metals also seems to play an important role in the emission of ligand-stabilized gold nanoclusters (AuNCs) of a few nm size (up to 2.7 nm)^25^ or silver nanoclusters (AgNCs).^26,27^ In these cases, the authors describe the photoluminescence as due to a ligand to metal–metal charge transfer (LMMCT), where excited electrons in the oxygen atoms of the ligands are transferred to the metal-oxide shell and subsequently to the metal core.

The comparison of standard semiconductor QDs and metallic NCs (less than 2 nm) reveals the fundamental differences in their mechanisms. Semiconductor QDs emit based on quantum confinement effects on a continuous band structure, resulting in a size-tunable bandgap. In contrast, metallic nanoclusters exhibit molecule-like behaviour, characterised by discrete HOMO–LUMO-type electronic energy levels rather than a band structure, as proposed by Ziefuss *et al.*^28^ Semiconductor QDs have been shown to exhibit a higher QY than metallic photoluminescent nanoclusters (50–90% for semiconductor QDs^29^ and 1–2% in the case of bare metallic nanoclusters^28,30,31^). However, the latter have the significant advantage of being able to avoid the use of toxic materials (commonly used Cd and Pb), thus rendering them suitable for bio-related applications due to the lower intrinsic toxicity of the metal used as the base.

The study of NCs obtained by PLAL is of particular interest. The extant literature on the subject is extremely scarce, with only a small number of works discussing the PLQY of this material.^28,30,31^ These findings are consistent with the literature, which reports PLQY values ranging from 1% to 2%.

Within this wide panorama, an emerging class of noble metal photoluminescent nanoclusters are AuNCs or AgNCs prepared by LSPC.^7^ In this case, photoluminescent nanoclusters are obtained either by pulsed laser ablation in liquid (PLAL) of the metal target^32,33^ or by the so-called laser fragmentation in liquid (LFL) process of preformed nanoparticles (NPs).^28,33^ Scaffardi *et al.*,^32^ used PLAL at fs laser pulses for the fabrication of polydisperse AgNCs with photoluminescence in the ultraviolet (UV) region, and interpreted the emission based on the Jellium model, with a HOMO–LUMO transition characteristic of a few atom systems (*N* < 10). Barcikowski *et al.*^28^ instead used LFL with ns laser pulses to fabricate AuNCs with emission in both the UV and visible (blue) regions. This is the first report where the oxidation state of the laser-synthesized AuNCs was considered in the theoretical model of the electronic properties of the NCs, modeled as Au_38_(OH)_24−*x*_(O)_*x*_^*x*−^ (*x* = 0, 2, 4) species. The model well described the absorption properties of the smaller clusters with the UV emission, still attributed to a HOMO–LUMO transition, but does not apply to the larger AuNCs with visible emission, which exhibit a photoluminescence up to a dimension of about 2.5 nm. Recently, our group demonstrated for the first time the possibility of performing a pulsed-laser-driven CO_2_ reduction reaction to control QY of organometallic gold-based NCs with luminescence in the blue region.^33^ In this case, it was observed that the organic material produced *in situ* allows the control of the QY but does not affect the spectral position of the emission, which was still attributed to a HOMO–LUMO transition, as observed for AuNCs synthesized by LFL.

In consideration of the sensitivity of the NCs optical properties to the specific experimental configuration, the integration of machine learning methodologies to guide the experimental design process of PLAL-based NC synthesis is a promising trend. These tools have the capacity to systematically manage the intricate parameter space of PLAL, with the primary objective of identifying the optimal energy-nanostructure balance. This is of paramount importance for enhancing material precision and yield. These issues remain significant challenges in the fabrication of nanostructures by LSPC techniques, a strategy that has been demonstrated to be effective in related laser-material processing studies.^34^

The scarcity and dispersion of literature data on photoluminescent NCs prepared by LSPC—due to varying experimental configurations—motivated us to perform a systematic investigation of the optical properties of ligand-free luminescent NCs of silver and gold, both obtained by PLAL with ns-laser pulses at a argon–water interface. This configuration has been chosen to eliminate the possible contribution of organic ligands, which, although in undetectable concentrations, may still be produced during the pulsed laser-driven CO_2_ reduction reaction (CO_2_RR) in water at equilibrium with the air environment, eventually influencing the optical behavior of the produced NCs. For both materials, colloidal dispersions of NCs without LSPR peaks were obtained by mechanical centrifugation, removing the plasmonic NPs, as reported in other works in the literature.^15,28,32,33^ After characterizing the structural, elemental, and morphological properties of the NCs, we investigated their optical properties in terms of spectral emission, bandgap, quantum yield, and lifetime. The similarities and the differences observed in the optical emission of the silver and gold nanoclusters are finally interpreted considering their size distribution and valence-states, suggesting that LSPC at the argon–water interface is a versatile tool to produce ligand-free noble metal NCs with controlled optical emission.

## 2 Materials and methods

### 2.1 Synthesis of ligand-free noble metal nanoclusters

Gold and silver colloidal suspensions containing plasmonic nanoparticles (NPs) and nanoclusters (NCs) were synthesized by PLAL using a Nd:YAG laser source operating at 532 nm, with a pulse duration of 5.8 ns and a repetition rate of 10 Hz.

The laser beam was focused onto a high-purity metallic target (Au or Ag, 99.99% purity, Kurt&Lesker – U.S.A) immersed in 5 mL of ultrapure water (UPW, 18.2 MΩ cm). The ablation was performed for 3 h in a sealed beaker where we created an argon–water interface by fluxing pure argon at a pressure of about 1.2 Atm. In particular, the ablation chamber was first evacuated until the liquid started to degas, followed by three argon flushing cycles with 1 h intervals at a pressure of 1.2 atm, to ensure the best removal of air from both the gaseous and the liquid phase. The fluence was calibrated at the value of approximately 80 J cm^−2^ using a software based on the ABCD matrix method, as reported in detail in ref. 33. After synthesis, NaOH was added to the colloidal dispersion to reach a final concentration of 4 mM, improving the stability of the suspensions. The separation of the ligand-free noble metal NCs from the plasmonic NPs was achieved through a three-step centrifugation process at 13.000 rpm and 0 °C, for a total duration of 3 h, utilising the freshly synthesized mixed colloidal suspension, reaching a final concentration in the range of 60 ppb. The procedure was done by adding the mixed suspension to a 2 mL microtube at each stage. Subsequent to each 1 hour centrifugation procedure, the upper layer was collected, thereby leaving the larger NPs. In the final stage, the presence of a colorless suspension was observed, indicating the successful removal of larger plasmonic NPs. The samples were stored protected from light in a standard refrigerator (4 °C), in a 2 mL microtube, without any specific procedure during transport.

### 2.2 Morphological and structural characterization

#### 2.2.1 Transmission electron microscopy

Morphological and structural characterization was performed by transmission electron microscopy (TEM) using a JEOL JEM-2100F microscope operating at 200 kV. The set of plasmonic NPs and photoluminescent NCs samples were prepared by drop-casting diluted colloidal dispersions onto carbon-coated copper or nickel grids and air-drying at room temperature. For each set of samples, at least 100 NPs or NCs were measured, and an average between the longest and smallest axes was used to build the size-distribution histograms, fitted with log-normal functions by using ImageJ and OriginLab software. High-resolution TEM (HRTEM) images were analyzed using Fast Fourier Transform (FFT) to determine interplanar spacings and identify crystalline phases.

#### 2.2.2 X-ray diffraction

Powder X-ray diffraction (XRD) patterns were recorded *via* an Oxford Diffraction SuperNova single-crystal diffractometer (Agilent) equipped with a Titan CCD detector and Cu Kα radiation (=1.5416 Å). Samples were obtained by drying the mixed colloidal dispersions containing plasmonic NPs and photoluminescent NCs under vacuum and were affixed to nylon loops for measurement. Data were collected over a range of 6.5–125° at 50 kV and 0.8 mA, with a step size of 0.0263°. Phase identification was performed using the QualX software (IC-CNR, Bari, Italy). The observed diffraction peaks were compared with reference data from the ICDD (International Centre for Diffraction Data) database to assess phase purity.

#### 2.2.3 X-ray photoelectron spectroscopy

X-ray photoelectron spectroscopy (XPS) measurements were performed using a VersaProbe II (ULVAC-PHI) spectrometer equipped with an Al K source (1486.6 eV) operating at 25.2 W, with both the source–analyzer and sample–analyzer angles fixed at 45°. The mixing colloidal suspension containing plasmonic NPs and photoluminescent NCs were prepared for analysis by drop-casting successive 40 µL aliquots of the colloidal dispersions onto pre-cleaned glass substrates, followed by drying at 50 °C. Survey spectra identified the elements present, while high-resolution scans were acquired for the Ag 3d, Au 4f, and O 1 s regions. The spectra were processed and fitted using Casa XPS software with Shirley background subtraction and Gaussian–Lorentzian line shapes. Relative atomic concentrations were calculated from the integrated peak areas normalized by the corresponding sensitivity factors.

### 2.3 Optical and spectroscopic characterization

#### 2.3.1 Optical absorption and emission

UV-Vis spectroscopy was employed both to evaluate NPs and NCs optical properties and to assess their colloidal stability. Extinction (absorption) spectra were obtained using a Lambda 950 spectrophotometer (PerkinElmer, USA) equipped with a 3.5 mL quartz cuvette, with an optical path length of 1 cm. The spectral range was set between 200 and 800 nm. Preceding each measurement, the colloidal samples were exposed to an ultrasonic bath to ensure homogeneous dispersion.

Steady-state (PL) and time-resolved (TRPL) photoluminescence analyses were performed using a Horiba Jobin Yvon Fluorolog 3-21 spectrofluorometer equipped with NanoLED excitation sources (304 nm, 1.2 ns pulse width, 1 MHz repetition rate) in Time-Correlated Single-Photon Counting (TCSPC) mode. Emission and excitation spectra were collected using quartz cuvettes (1 cm path length), maintaining sample extinction below 0.1 to minimize reabsorption.

Photoluminescence Quantum Yields (QY) were determined *via* a comparative method using Rhodamine 101 (QY = 100%) as a reference. Luminescence decay curves were fitted using a double-exponential model to extract fast (*τ*_1_) and slow (*τ*_2_) lifetime components.

#### 2.3.2 Surface-enhanced Raman spectroscopy

Surface-enhanced Raman spectroscopy (SERS) was performed working with a confocal micro-Raman spectrometer (XploRA, HORIBA, Japan) equipped with a thermoelectrically cooled CCD detector operating at −50 °C. The excitation source at 638 nm was focused onto the sample surface through a 100× objective lens, producing a spot of approximately 10 µm in diameter. The laser irradiance was maintained below 10^−2^ kW mm^−2^ to prevent photoinduced alterations of the samples. Measurements were performed on glass slides using the drop-casting method, where six 20 µL aliquots of the colloidal dispersion were deposited and air-dried, as previously reported in ref. 35.

## 3 Experimental results and discussion

### 3.1 Morphological and structural characterization

The samples were divided into two sets: Ag-based and Au-based nanostructures. The synthesis procedure employed for both targets was analogous, with the use of water, the selected target (Au or Ag), and a laser beam as the exclusive elements. The inert atmosphere, which was maintained using argon, was employed to ensure the attainment of fully inorganic nanostructures, thereby eliminating the possibility of interaction with carbonic gases, as previously described elsewhere.^33,36^

**Fig. 1:**
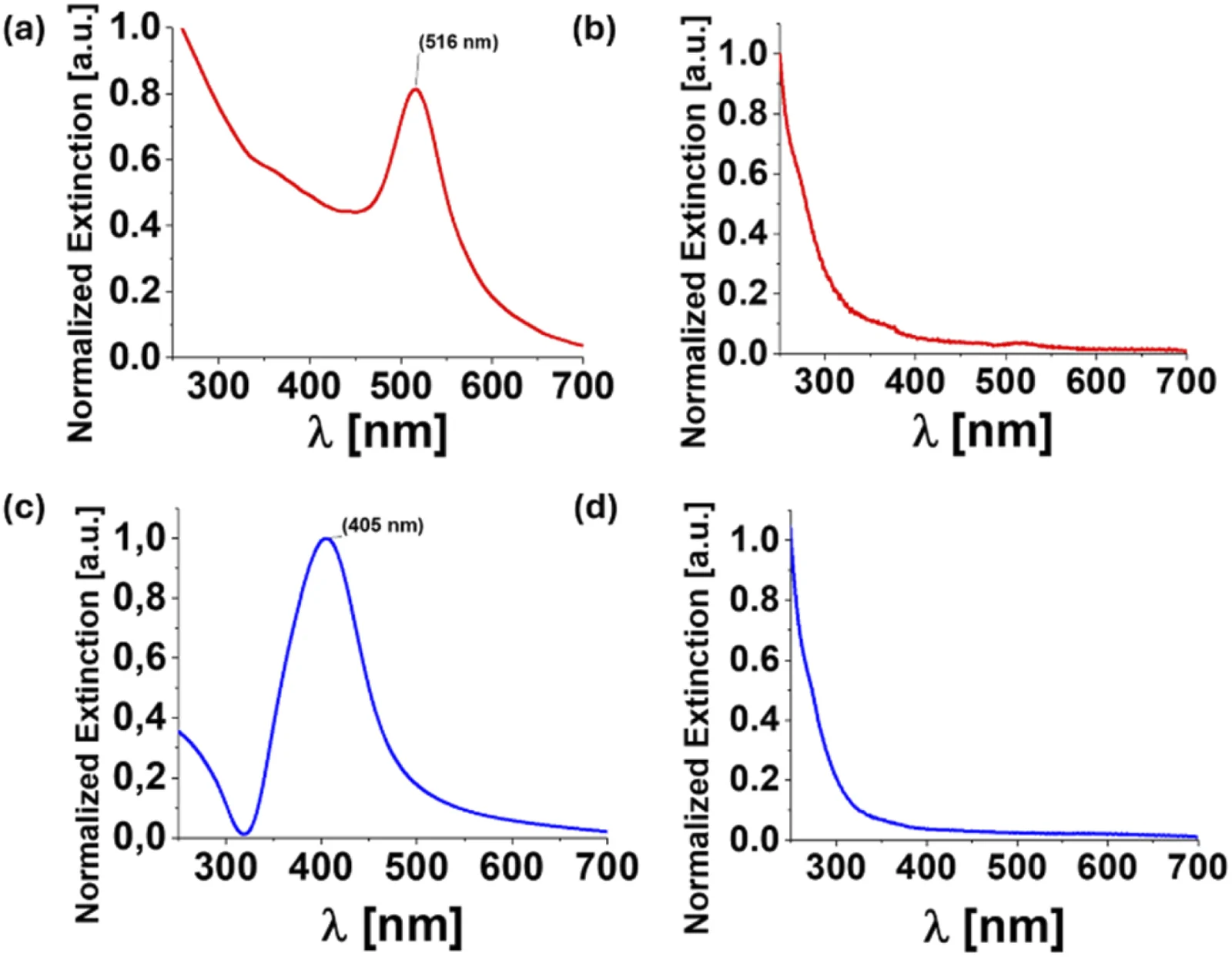
Extinction spectra of Au (red, a and b) and Ag (blue, c and d) before and after bleaching (centrifugation). The plasmonic bands of freshly synthesized NPs (a and c) are absent in the NC fractions (b and d), indicating the removal of larger plasmonic nanostructures.

The freshly synthesized mixed colloidal suspension of Au (containing larger plasmonic NPs and smaller NCs) did not consist solely of spherical nanoparticles; instead, it exhibited irregular nanostructures formed by coalescence of smaller NPs. This coalescence broadens the characteristic extinction profile up to 700 nm (Fig. 1a)^8,37^ suggesting increased dipolar interactions and agglomeration within the colloidal dispersion. This phenomenon suggests an increase in the dipolar interaction within the colloidal dispersion, which ultimately results in agglomeration. As demonstrated in Fig. 1a, the standard LSPR peak associated with plasmonic gold nanoparticles in suspension is observed at 516 nm, compatible with a size distribution in the range of 8 to 15 nm.^15,33,35,38^ A similar effect is observed in the case of plasmonic AgNPs (Fig. 1c), with the extinction profile broadening up to 600 nm, suggesting once again a high degree of interaction between nanoparticles in suspension and the tendency to agglomerate. The LSPR characteristic peak is observed at 405 nm, as is typically observed in the literature for size distributions in the range of 20 nm.^39–42^

Following the centrifuge bleaching protocol, the resulting ligand-free photoluminescent NCs exhibited no LSPR band in their extinction spectra (Fig. 1b–d), consistent with the removal of larger plasmonic nanoparticles capable of supporting LSPR.^28,43^

**Fig. 2:**
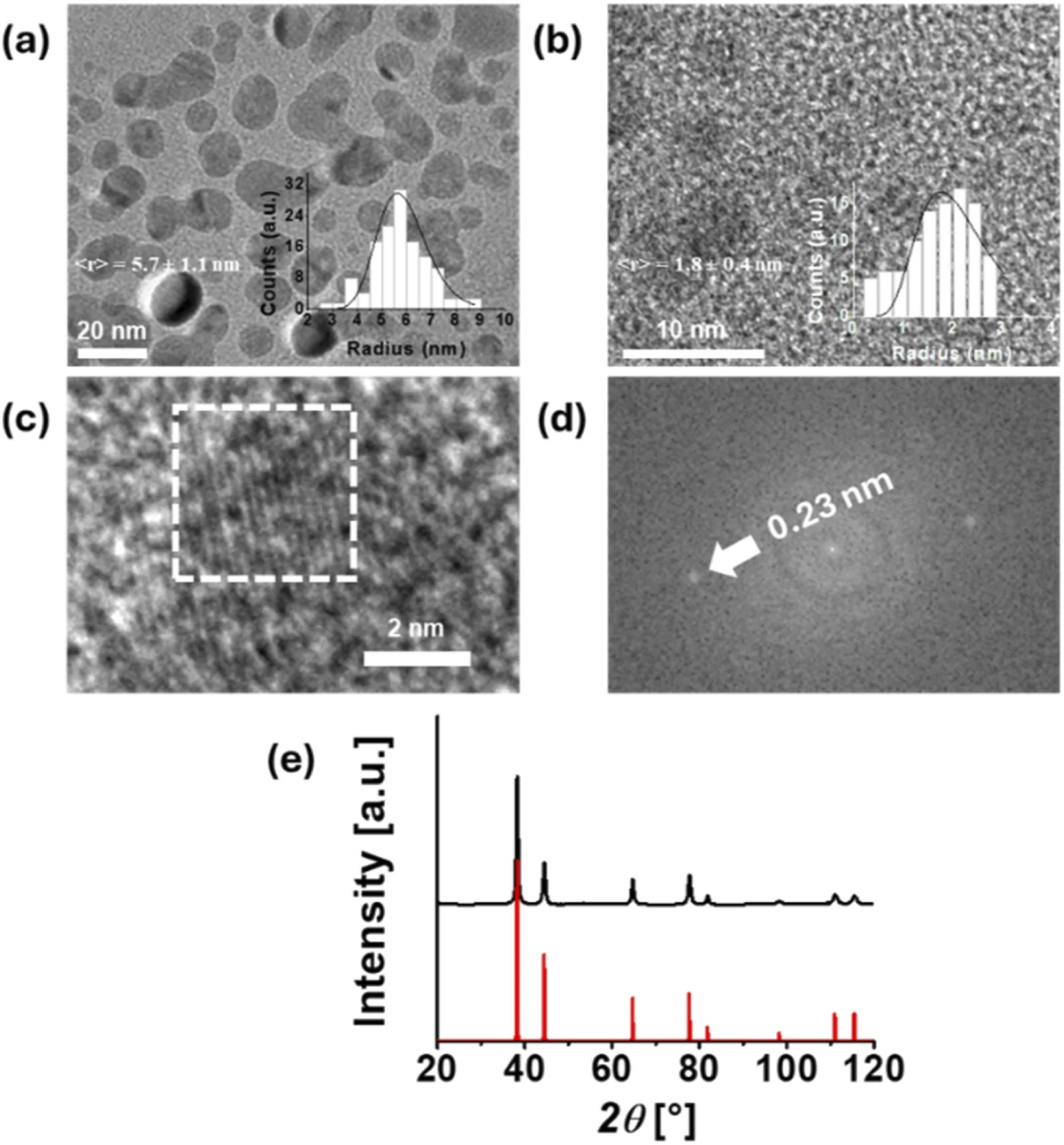
TEM characterization of the ligand-free AuNPs (a) and AuNCs (b). The insets show the corresponding size-distribution histograms. (c) High resolution TEM (HRTEM) image of AuNPs and (d) Fast Fourier Transform (FFT) pattern from the HRTEM image. In panel (e), the black line shows the XRD pattern of dried, as-synthesized Au mixed colloidal suspension containing NPs and NCs, and the red line shows the Au reference (ICDD 00-001-1172).

Particle sizes were estimated from TEM images by determining the maximum projected dimension of single NPs. We obtained a mean radius of approximately 5.7 nm for Au NPS with a broad size distribution (±1 nm), as represented in Fig. 2a. Such heterogeneity is characteristic of PLAL-generated colloids in low-ionic-strength media, where limited electrostatic stabilization promotes coalescence and agglomeration as observed in the extinction spectra (Fig. 1a).^28,44^ The AuNCs obtained by the centrifugation bleaching process exhibited a mean radius of about 1.8 nm (see Fig. 2b). The absence of a detectable LSPR band in our 3.5 nm AuNPs highlights a unique structural consequence of the PLAL process. For ideal single crystals, the theoretical limit for plasmon survival due to quantum confinement is approximately 2.5 nm.^45^ However, at larger sizes, several factors can suppress the LSPR: surface oxidation dampens electron oscillation and reduces plasmon intensity,^46–48^ while the ultra-fast thermal quenching inherent to laser ablation can freeze nanoparticles into a polycrystalline state, introducing structural defects that further disrupt the collective electron response.^49^ The resulting high density of internal grain boundaries introduces an extreme grain boundary scattering mechanism.^50^ This not only suppresses the collective electronic oscillation *via* severe plasmon damping but also may artificially shifts the plasmon-to-molecular transition to a larger geometric size regime compared to chemically synthesized counterparts.^43^

Fig. 2c presents an HRTEM image of a representative crystalline structure of AuNC, where FFT analysis (Fig. 2d) reveals interplanar spacings of 0.23 nm, corresponding to the crystalline (200) plane of the metallic gold.^51,52^

A comparison of the experimental XRD pattern with the standard X-ray reference of metallic gold (ICDD 00-001-1172) (Fig. 2e – red pattern) reveals the absence of oxide-related signal traces. All observed peaks match the reflections of metallic gold, with the most intense peaks at 2*θ* = 38.3° and 44.5°, corresponding to the (111) and (200) planes (*d* = 0.23 nm and 0.20 nm, respectively). The presence of the (111) plane was further corroborated by HRTEM imaging.^53^

**Fig. 3:**
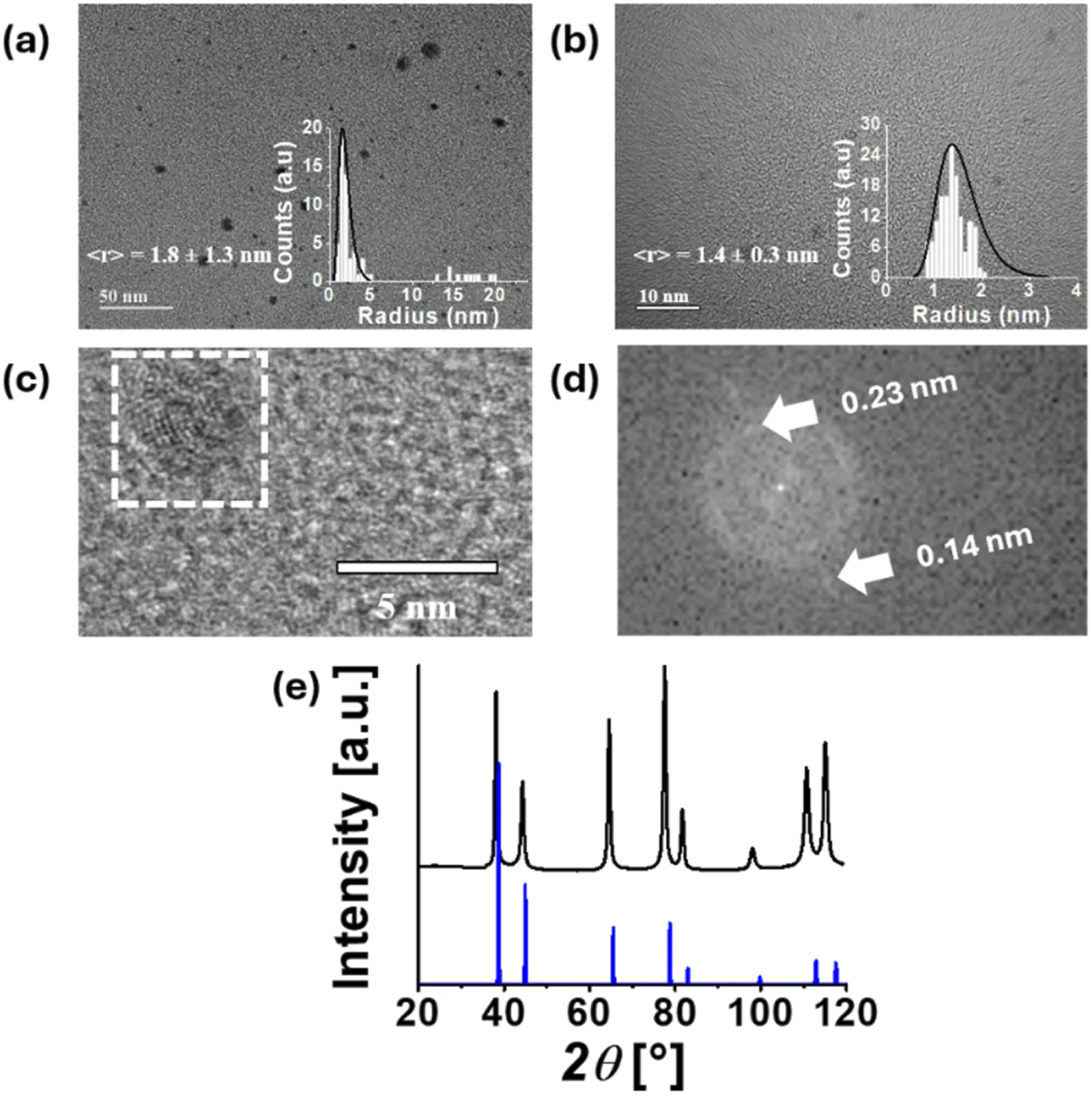
TEM characterization of the ligand-free AgNPs (a) and AgNCs (b). The insets show the corresponding size-distribution histograms. (c) High resolution TEM (HRTEM) image of AgNPs and (d) Fast Fourier Transform (FFT) pattern from the HRTEM image, showing the crystalline planes of the silver lattice. In panel (e), the black line shows the XRD pattern of dried, as-synthesized Ag mixed colloidal suspension containing NPs and NCs, and the blue line shows the Ag reference (ICDD 04-0783).

On the other hand, silver NPs have a spherical morphology, but differently from the AuNPs, two populations were observed (Fig. 3a), with a population centered at 1.8 nm and a very dispersed distribution around 15–20 nm. The LSPR peak of plasmonic AgNPs corresponds to the size distribution of the larger population, consistent with TEM observations.^42^

This behavior agrees with previous reports on PLAL-derived AgNPs, where agglomeration occurs rapidly upon contact with water.^54^ Moreover, the LFL effect caused by laser pulses at 532 nm wavelength is less efficient for AgNPs than for AuNPs.^55^

In accordance with the bleaching protocol employed for the selection of the size of the AuNCs, the process was also followed to get ligand-free AgNCs, which exhibited an average radius of approximately 1.4 nm (see Fig. 3b). As shown by the UV-Vis spectra, the plasmonic band centered at 405 nm in the freshly synthesized plasmonic AgNPs disappears in the ligand-free AgNCs obtained after the bleaching process (Fig. 1d).

HRTEM analysis revealed well-defined domains (Fig. 3c), which, by FFT patterns (Fig. 3d) evaluation, exhibited interplanar spacings of approximately 0.23 nm and 0.14 nm, corresponding to the (111) and (220) planes of FCC metallic silver (JCPDS 04-0783) (Fig. 3e, blue line).^56^ Notably, these values partially overlap with those of Ag_2_O and AgO, limiting the ability of HRTEM to unambiguously distinguish between metallic and oxide phases.

However, the analysis of the XRD patterns suggests that the crystal portion of the AgNPs is predominantly metallic. The diffractogram of the dried mixed Ag colloidal suspension (Fig. 3e, black line) shows reflections at 2*θ* = 38.7° and 45°, corresponding to the (111) and (220) planes of metallic Ag, with spacings of 0.23 nm and 0.14 nm, respectively. No crystalline silver oxide phases were detected within the sensitivity limits of the instrument.

In summary, TEM, HRTEM, and XRD provide evidences that the AuNCs and AgNCs synthesized under inert conditions consist of crystalline metallic structures without detectable organic species.

### 3.2 Surface chemical analysis

The chemical composition of the ligand-free noble metal NPs was investigated using SERS and XPS to look for noble metal-carbonyl and oxide species, respectively.

**Fig. 4:**
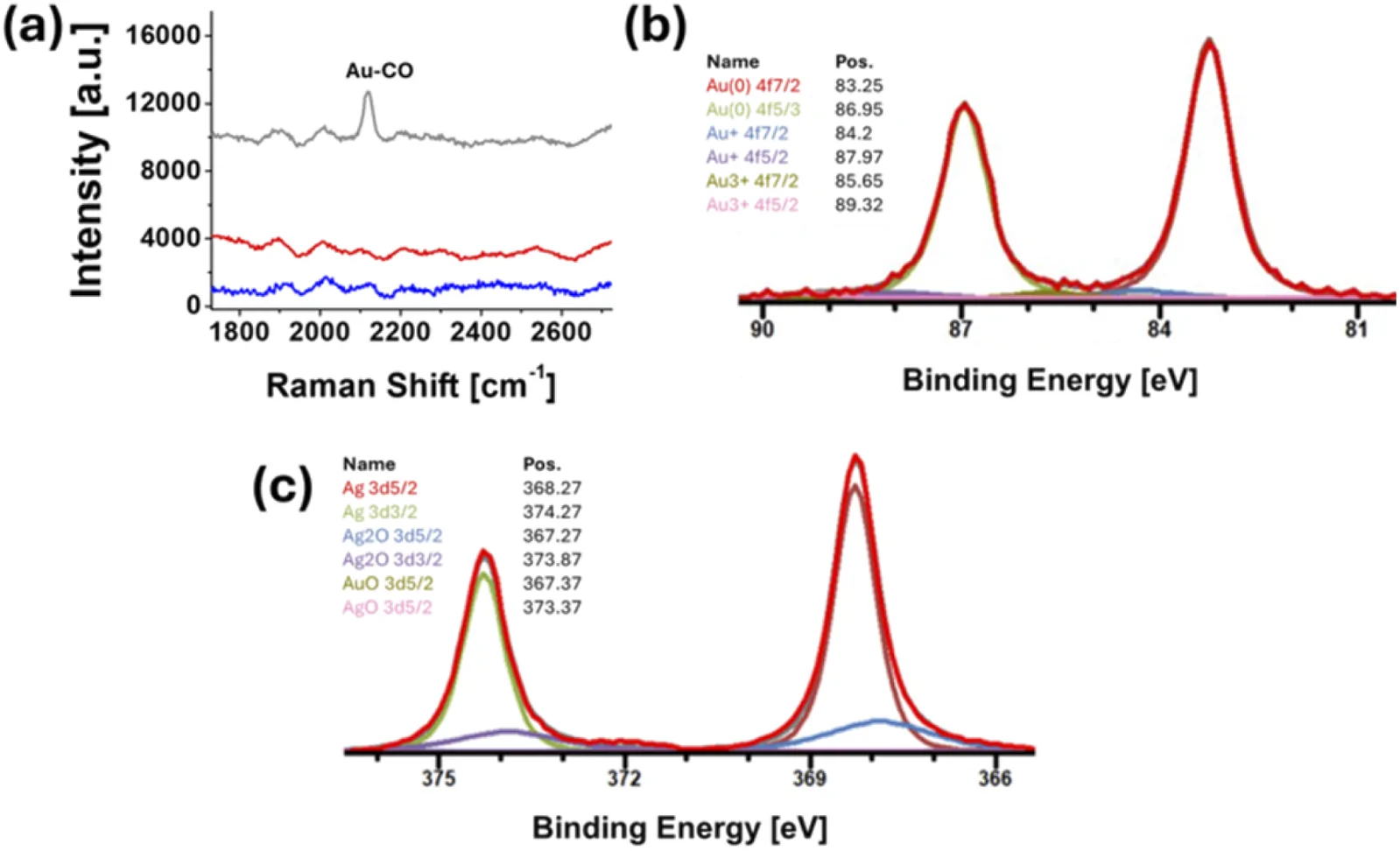
(a) SERS spectra obtained from PLAL-synthesized ligand-free AuNPs (red) and AgNPs (blue) prepared at the argon–water interface, and from carbon monoxide-rich gold nanoparticles prepared at the CO_2_–water interface (gray; adapted from ref. 57). (b) XPS spectrum of the Au 4f core level of ligand-free AuNPs, showing peak deconvolution into Au^0^, Au^+^, and Au^3+^ contributions, with the corresponding binding energies indicated in the inset. (c) XPS spectrum of the Ag 3d core level for ligand-free AgNPs, showing peak deconvolution into Ag^0^, Ag_2_O, and AgO contributions, with corresponding binding energies indicated.

SERS results for the AuNPs (Fig. 4a – red line) show no peaks in the range 1600–2600 cm^−1^, suggesting that no Au-carbonyl species are formed by PLAL at the argon–water interface, in contrast with recent results reporting the synthesis of carbon monoxide-rich gold nanoparticles (COR-AuNPs) by PLAL at the CO_2_–water interface, where a clear peak of gold-carbonyl species centered at 2120 cm^−1^ is observed.^33,57^

XPS measurements of the Au 4f core level (Fig. 4b) revealed that the Au-based nanostructures are predominantly metallic, with negligible oxide contributions. The Au^0^ doublet was clearly observed at 83.74 eV (4f_7/2_) and 87.41 eV (4f_5/2_). Two minor additional components, assigned to Au^+^ and Au^3+^, are visible at 84.64/88.31 eV and 86.14/89.81 eV, respectively. Although the oxidized species represent only a minor contribution of approximately 4%, their presence cannot be excluded, as it is possible to observe the vanishing of AuNP LSPR after the bleaching process, meaning a possible large contribution of oxide species.^58^

This assignment is consistent with previous reports of oxidized gold species formed during PLAL of gold targets in water, where the fraction of gold oxide typically ranges from 3% to 7%.^44,59,60^

XPS measurements were performed on the freshly synthesized mixed colloidal suspensions due to the low mass yield of the isolated NC fraction after centrifugation, which restricted direct XPS film deposition. As demonstrated by Ziefuss *et al.*,^28^ ultrasmall gold nanoclusters can exhibit an oxidized species fraction reaching up to 30%, a value significantly higher than that observed on the surface of larger nanoparticles. Following this rationale, it is reasonable to assume that the degree of oxidation in the isolated NC fraction is higher than in the larger NP population for both Au and Ag systems. Consequently, the oxidation levels determined here (∼4% for Au and ∼20% for Ag) represent a lower limit for the oxidation state of the isolated photoluminescent NCs. While techniques such as X-ray Absorption Near Edge Structure (XANES) spectroscopy would be required to precisely assess the oxidation state of sub-2 nm clusters, our XPS results provide a reliable baseline that indicates the ligand-free nature of the colloids and highlights silver's higher oxidative susceptibility compared to gold under inert argon–water conditions.^51^

In this case study, the results indicate that rather than forming a distinct, phase-separated core–shell heterostructure, surface oxidation/hydroxylation in these ultrasmall nanoclusters introduces an atomically thin, passivating surface layer (metal^*δ*+^ –O/OH). These surface species may play a decisive role in defining the photoluminescence peak positions by introducing discrete electronic states within the quantum-confined energy gap. Upon optical absorption, photoexcited charge carriers relax into these localized surface-state levels *via* Oxygen-to-Metal Charge Transfer (OMCT) or Ligand-to-Metal Charge Transfer (LMCT) interactions.^61,62^ Radiative recombination from these charge-transfer states down to lower-lying core electronic levels governs the observed emission wavelengths and dictates the characteristic Stokes shift. Thus, surface oxides act as active electronic modulation centers that dictate emission energy and carrier dynamics, rather than a passive structural shell layer.

Similar to Au nanostructures, the SERS spectra of Ag nanostructures (Fig. 4a -blue line) showed no vibrational features associated with Ag–CO species. Notably, these species exhibit a Raman response similar to that of gold–carbonyl species.^63,64^ This finding suggests that PLAL of silver targets at the argon–water interface also results in the formation of fully ligand-free AgNPs. This phenomenon can be attributed to the inability of the pulsed laser-driven CO_2_ reduction reaction to occur within this environment.^33,57^

XPS analysis of the Ag 3d core level (Fig. 4c) indicates that the nanoparticle surface is predominantly metallic. The Ag^0^ component, located at 368.27 eV (3d_5/2_) and 374.27 eV (3d_3/2_), constitutes the majority of the signal. A weak shoulder at 367.87 eV and 373.87 eV, reflects the presence of a thin oxide layer. The relative peak contributions (≈80% Ag_0_ and ≈20% Ag_2_O + AgO) are in line with the mild surface oxidation commonly observed in PLAL-produced AgNPs in water.^65,66^ The apparent discrepancy between XRD and XPS can be explained by a thin silver oxide passivating layer, possibly amorphous, consistent with the LSPR disappearance in larger plasmonic AgNPs (Fig. 1d).^30^ The presence of such a layer can be detected by XPS in the case of silver, given its lower oxidative barrier in comparison to gold, and the subsequent resultant increased oxidation.^67^ In contrast to XRD, which is characteristic of bulk analysis, XPS probes only the first few nanometers of the sample. In this sense, the absence of crystalline silver oxide reflections in XRD is consistent with the observed XPS signals.

Overall, SERS and XPS analyses consistently show that both Au and Ag nanostructures produced at the argon–water interface are fully inorganic, composed of metallic crystalline cores surrounded by only a few percent of a superficial oxide species, with no detectable organic contamination. The most significant discrepancy arises from the oxidation level, wherein gold exhibits approximately 4% oxidized species, while silver displays approximately 20%, underscoring the pivotal role that the target composition and synthesis environment play in determining the phase of the noble metal nanostructures.

It is important to contextualize the diagnostic scope and limitations of SERS and XPS in evaluating the purity of these colloids. In our experimental setup, SERS served primarily as a highly sensitive tool (capable of detecting sub-picomolar surface species) to monitor the metal-carbonyl vibrational band at 2120 cm^−1^, which marks CO_2_RR-induced species during PLAL in water containing dissolved CO_2_.^33,57^ The total absence of this feature suggests that no laser-driven chemical reduction of dissolved carbon gases occurred under the argon atmosphere.

However, SERS does not exclude trace non-resonant organic impurities potentially acquired during ambient post-synthesis handling. On the other hand, XPS was mainly utilized to determine the elemental oxidation states of the noble metal nanoparticles. Regardless of the XPS detection limit, the ubiquitous presence of adventitious carbon (C1s at 284.8 eV) from atmospheric exposure prevents its use as a decisive tool for isolating trace environmental contaminants from synthesis-related organic ligands. Therefore, the ligand-free nature of the nanostructures is supported by combining the controlled, precursor-free ablation environment (argon–water interface) with the SERS-confirmed absence of *in situ* synthesized metal-carbonyl complexes.

### 3.3 Optical properties

**Fig. 5:**
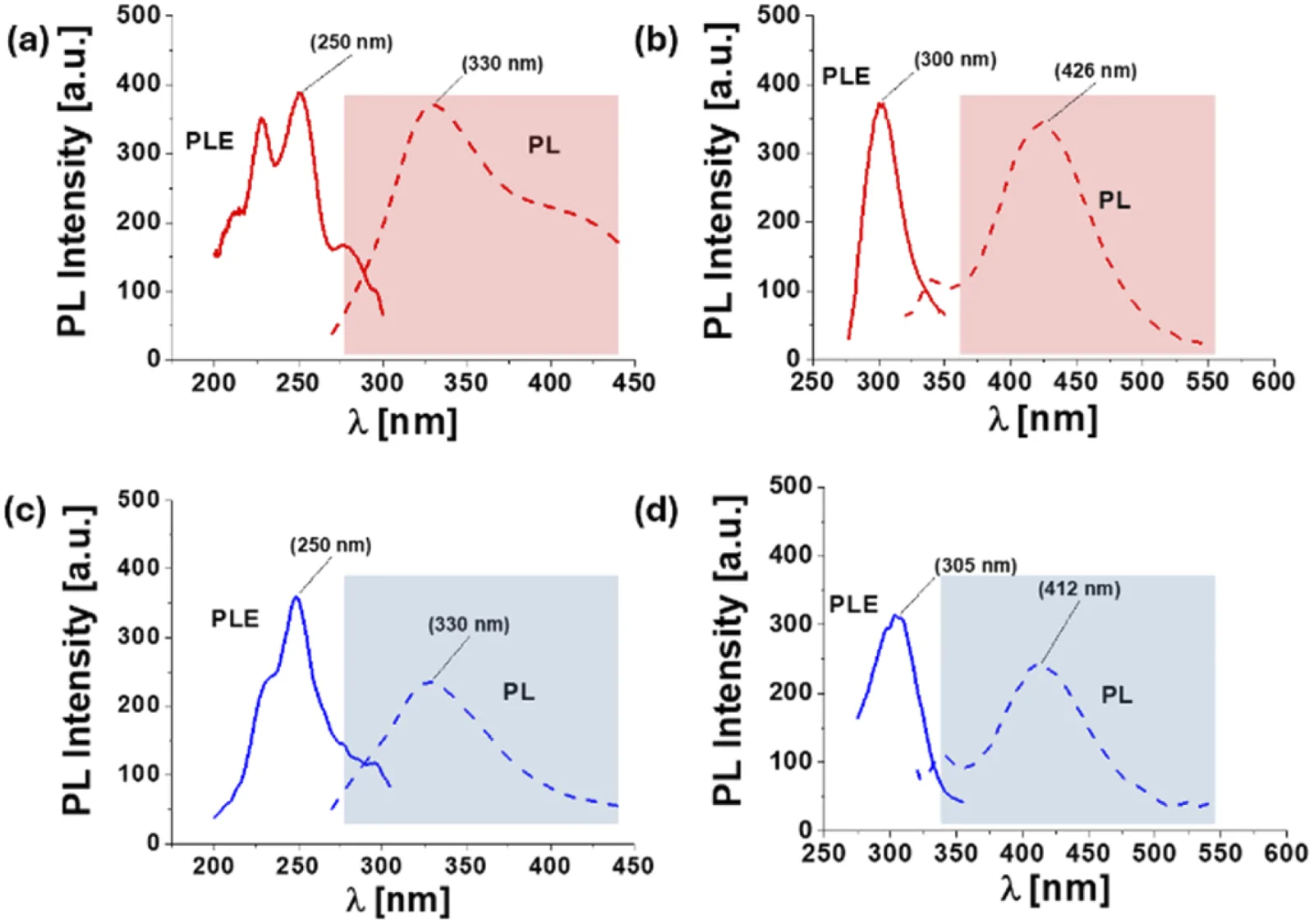
Steady-state photoluminescence excitation (solid lines) and emission (dashed lines, highlighted) spectra of ligand-free AuNCs (red) and AgNCs (blue) synthesized by PLAL at the argon–water interface. Both materials exhibit UV emission under 250 nm excitation (a and c; ≈330 nm) and blue emission under ≈304 nm excitation (b and d; 426 nm for Au, 412 nm for Ag), with corresponding excitation maxima matching the respective excitation wavelengths.

Fig. 5 presents the photoluminescence excitation (PLE) and photoluminescence emission (PL) spectra of the ligand-free noble metal NCs. Both materials exhibit two well-defined emission bands, depending on the excitation wavelength: an intense band at the near-UV, about 330 nm is present for 250 nm excitation for both Au and Ag samples (Fig. 5a–c); another intense band is present at the blue, about 426 nm for 300 nm excitation for Au and at 412 nm for 305 nm excitation for AgNCs (Fig. 5b–d), findings consistent with those reported in the extant literature concerning noble metal nanoclusters of a similar size.^31–33^ These two last bands, although slightly shifted, appear very similar in shape. The principal distinction is that for AuNCs, this band is also evident under 250 nm excitation, as evidenced in the PL spectra of Fig. 5a, where a discernible shoulder of the PL is present at approximately 430 nm.

PLE spectra for UV emission exhibit two main peaks (approximately 227 nm and 250 nm) for both species, although for Ag samples, the first peak appears as a shoulder rather than a distinct peak.

The occurrence of two PLE peaks can be attributed to the presence of different absorption centers in ultrasmall noble metal nanoclusters. They can also be attributed to different sp–sp intraband transitions across the HOMO–LUMO gap.^68^ Metal nanoclusters are known to exhibit different excitation centers depending on several factors: surface groups related to pH variation,^69^ dopants incorporated into the nanocluster structure,^70^ defects and oxidized states,^71^ and contributions from different cluster sizes.^28^ In this study, the smaller nanoclusters are likely the main responsible for this absorption at a higher energy level (220 nm) attributed to the overlap of different sp–d band transitions,^68^ rather than to ligand or donor–acceptor effects, as no secondary products are observed in the samples.^72^ Notably, no other chemical species were used during synthesis, suggesting that the observed effects are not attributable to surface chemical groups.

Unlike atomically precise, ligand-monolayer-protected noble metal clusters that feature narrow molecular-like emission bands, the synthesized bare Au nanoclusters exhibit a broader PL emission spectrum. This spectral broadening is attributed to two main factors: (i) ensemble heterogeneity, where sub-populations of varying cluster sizes contribute to a convolution of size-dependent emission peaks, and (ii) strong involvement of localized surface states and surface oxidation (*e.g.*, Au–OH/Au–O species),^28^ which introduce a distribution of radiative recombination channels.

Although structural characterization indicates bulk-like fcc local order, the optical response shows no dependence on specific crystallographic planes. In this extreme quantum confinement regime, optical transitions are governed primarily by spatial boundary constraints and localized surface states—including surface oxidation/hydroxylation—rather than plane-dependent selection rules typical of larger, well-faceted nanostructures.

The optical QY at the blue spectral window was determined by a comparative method using Rhodamine 101 as a reference, resulting in approximately 1.2%, for AuNCs, a bit lower than reported for AuNCs synthesized by PLAL at the air–water interface (See SI).^28^ The QY of the UV band could not be reliably determined owing to the lack of appropriate standards in this spectral region. As for AgNCs, the QY at the blue spectral window was determined to be approximately 2%, a finding that is in accordance with the results reported in the literature concerning the synthesis of ligand-free noble metal nanoclusters by PLAL.^28,30,31^

**Fig. 6:**
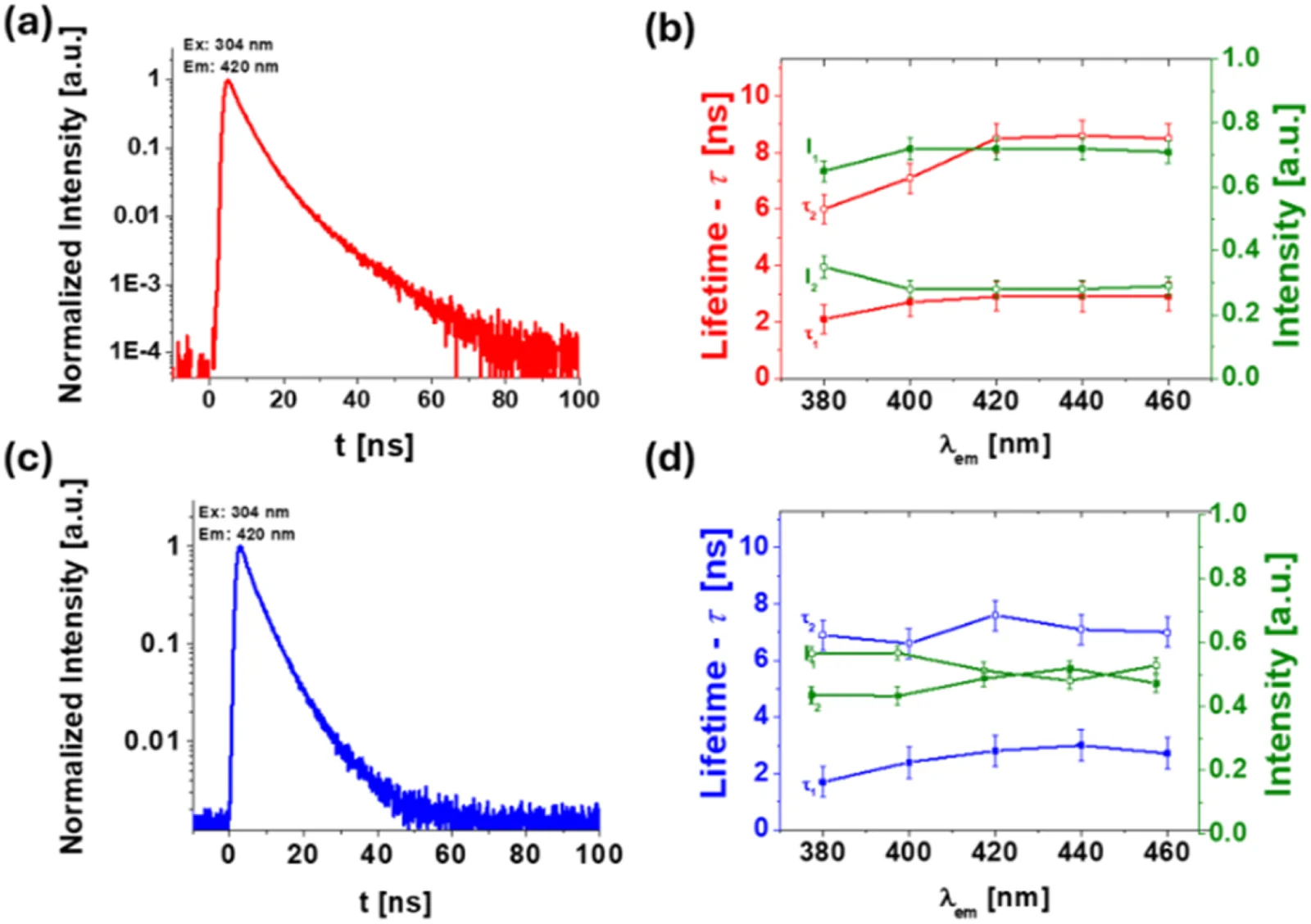
Photoluminescence decay profiles ((a) AuNCs, (c) AgNCs) and wavelength-dependent lifetime components ((b) AuNCs, (d) AgNCs) under 304 nm excitation (emission at 420 nm). All decays were fitted with a bi-exponential model.

TRPL measurements were performed to elucidate the photoemission mechanisms by analyzing the temporal decay dynamics of the emitted photons (Fig. 6). All decay curves were fitted using a bi-exponential model.

For AuNCs (Fig. 6a and b), the fast decay component *τ*_1_ ranges from 1.4 to 3.0 ns and dominates the emission dynamics, accounting for approximately 80% of the total intensity. This fast component is attributed to radiative sp–sp intraband transitions across the HOMO–LUMO gap in ultrasmall metallic clusters, consistent with the mechanism reported for sub-2 nm gold nanoclusters.^28^ The slower component, *τ*_2_ (6–9 ns, ≈20% contribution), is consistent with the secondary relaxation channel attributed above to surface oxide/hydroxide trap states. This heterogeneous trap-state population may additionally include contributions from surface structural defects, such as atomic vacancies and grain boundaries, which can arise from the rapid cooling rates characteristic of the PLAL process—a non-equilibrium synthesis route reported to favor the formation of metastable surface phases.^51,52,73–76^ Both the chemical (oxide/hydroxide) and structural (defect-related) origins would be expected to broaden the distribution of available surface states and reduce HOMO–LUMO orbital overlap relative to the core-localized transition, consistent with the longer effective decay time observed for *τ*_2_.^28^ Importantly, both lifetime components exhibit negligible wavelength dependence across the 380–460 nm emission window, indicating that emission originates from a single emitting state with homogeneously broadened spectral features rather than from an ensemble of spectrally distinct emitters with different sizes.^77^ This behavior is indicative of intrinsic core-state emission in quantum-confined metal nanoclusters.

AgNCs also exhibit a similar bi-exponential decay dynamics (Fig. 6c and d), yet their intensity contribution distribution patterns differ considerably. The fast component *τ*_1_ (1.7–3.0 ns) contributes 45–55% of the total intensity, while *τ*_2_ (6.5–7.5 ns) exhibits comparable weight. This observation indicates a more balanced contribution from the two decay channels in the AgNC system compared to the *τ*_1_-dominated decay in AuNCs. This finding leads to the proposal that the AgNC system may possess a greater density of surface trap states, introducing additional relaxation channels without substantially reducing light-emitting efficiency or altering the emission profile.^76^ This phenomenon is attributed to enhanced surface oxidation, as evidenced by XPS analysis of the as-synthesized colloidal suspension containing both AgNPs and AgNCs that revealed the presence of Ag_2_O/AgO. While direct XPS characterization of isolated AgNCs was not feasible due to limitations in sample preparation, it is reasonable to infer that surface oxidation extends to the nanocluster fraction, and given the higher susceptibility of silver to ambient oxidation compared to gold, the oxidation of the AgNCs surface can be higher, which is in alignment with the larger full width at half maximum (FWHM) observed for the AgNCs in the PLE measurement (Fig. 5d).

The LSPR suppression in both systems further indicates the presence of oxide species, which may damp electron oscillation, reduce delocalized electrons, and destabilize LSPR formation.^58,78^

Also, it has been demonstrated that surface oxidation modifies the photophysical properties of noble metal nanoclusters, resulting in alterations to both emission profiles and QY.^71,79^ The presence of such oxide-induced trap states would provide a plausible explanation for the increased relative contribution of *τ*_2_ in AgNCs. This can also be correlated with the larger PLE band of AgNCs, where the more oxidized surface slightly increases the optical absorption, as discussed below by Tauc plot analysis in the following section.

The characteristic timescales (1–9 ns) and spectral emission range observed for both AuNCs and AgNCs are consistent with, and can be rationalized by analogy to, the metal-nanocluster photoluminescence framework proposed by Ziefuß *et al.*^28^ In that work, DFT calculations on ligand-free, fully inorganic Au nanoclusters showed that the relevant HOMO–LUMO transition has mixed orbital character, with the HOMO weighted toward surface hydroxide/oxide contributions and the LUMO weighted toward the metallic core, and that the spatial overlap between these orbitals—tunable *via* surface charge and pH—influences the emission intensity. The same study also reported clearly distinguishable core- and surface-dominated emission contributions depending on particle size. By analogy with this reported behavior, we propose that the faster decay component observed in our PLAL-synthesized AuNCs and AgNCs is plausibly associated with the more core-weighted character of this transition, while the slower decay component is plausibly linked to surface oxide/AuO–AuOH-related states. This slower relaxation is consistent with the structurally and energetically heterogeneous nature of surface oxide/hydroxide trap states, which reduces HOMO–LUMO orbital overlap and the associated transition dipole moment, and can introduce additional non-radiative relaxation pathways prior to emission—both of which are expected to lengthen the effective decay time relative to the more core-localized transition.^28,52,76^ We emphasize that this assignment is presented as a literature-supported hypothesis rather than a fully established mechanism.

**Fig. 7:**
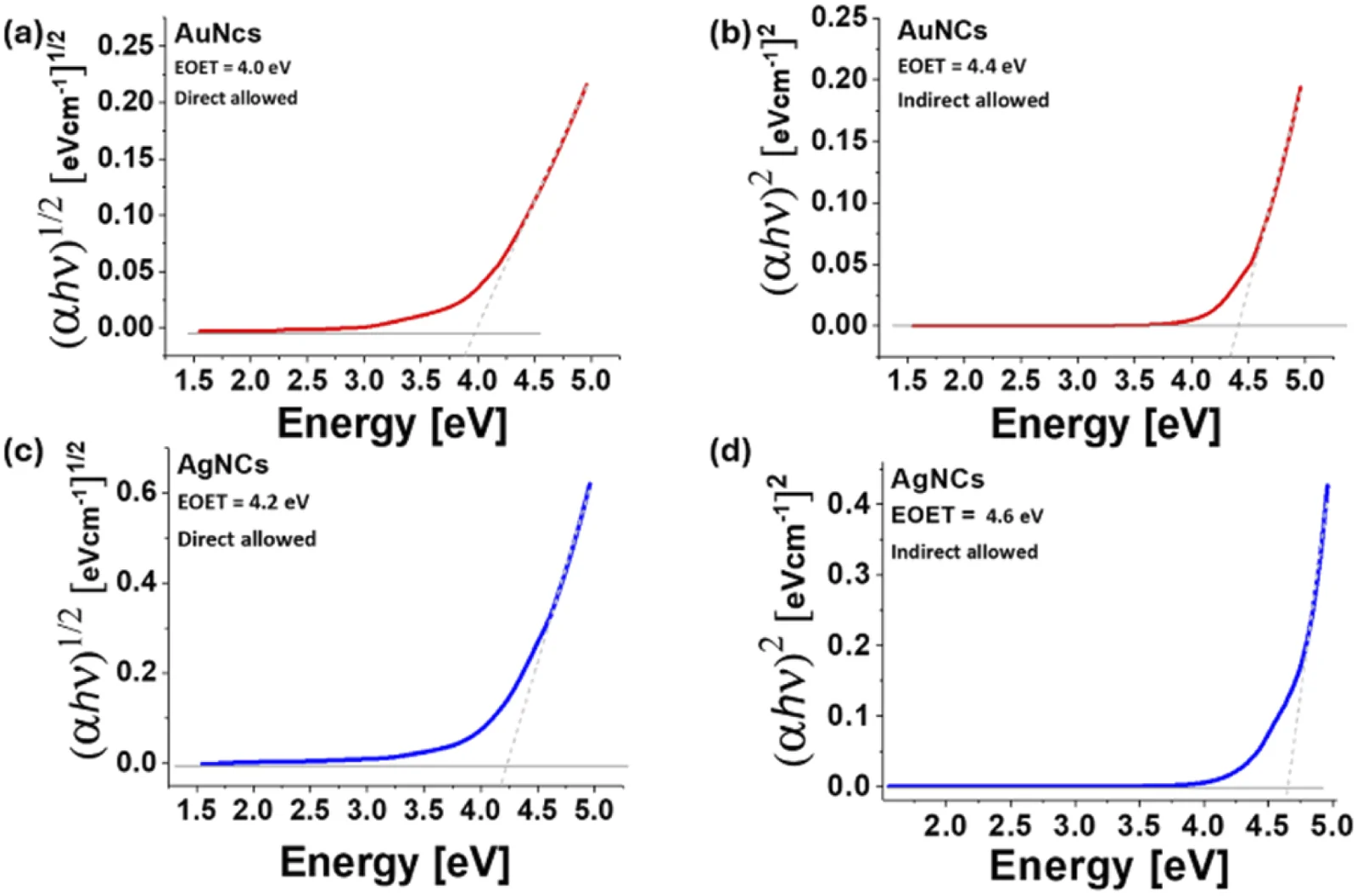
Tauc's plots for the determination of effective optical energy transition of fully inorganic, photoluminescent AuNCs (red) and AgNCs (blue). Direct allowed transitions are shown for AuNCs (a) and AgNCs (c); indirect allowed transitions are shown for AuNCs (b) and AgNCs (d). Dashed gray lines represent linear fits to the Tauc's plots.

The effective optical energy transition (EOET) of the photoluminescent AuNCs and AgNCs were estimated using Tauc's plots. For both materials, direct allowed fits yielded consistent values in the range of 4.0–4.6 eV (corresponding to 270–310 nm). This spectral region encompasses 300–305 nm, the excitation wavelength that maximizes the blue emission. Although the Tauc formalism was originally developed for extended semiconductors with continuous electronic energy bands, its application here serves strictly as a phenomenological tool to extract the threshold energy of discrete electronic excitations in quantum-confined metal clusters. Therefore, the extracted EOET values must be interpreted as effective optical energy transition (*i.e.*, the onset of discrete sp–sp or d–sp interband transitions) rather than conventional semiconductor band gaps. In these ultrasmall systems, the emergence of discrete molecular-like energy levels enables the determination of an effective transition onset even in the absence of a periodic lattice band structure. Notably, the photoluminescence lifetimes measured in the 1–9 ns range are consistent with radiative allowed transitions rather than strongly forbidden processes. In this context, the superior linearity obtained using the direct-allowed Tauc representation in Fig. 7a and c, compared to the indirect model, may indicate that the optical transitions possess significant dipole-allowed character. Moreover, in ultrasmall clusters, the loss of long-range translational symmetry relaxes the crystal momentum selection rules, making momentum conservation less restrictive and enabling transitions that effectively behave as direct excitations.

Notably, the effective optical energy transition values for AuNCs and AgNCs are nearly identical, and both materials emit in the near UV (330 nm for AuNCs, and 329 nm for AgNCs) and in the blue spectral window (426 nm for AuNCs, and 412 nm for AgNCs). This similarity in optical behavior occurs despite the different surface chemistries, as revealed by XPS analysis. The precursor AuNPs contained only a negligible fraction of oxidized species (approximately 4%), whereas the AgNPs exhibited a substantially higher oxidized fraction (approximately 20%). This suggests that surface states do not determine the photoluminescence mechanism primarily, but contribute discretely. Otherwise, a significant difference in emission energy and effective optical bandgap would be expected between the two materials, but this was not observed except for a slight blue shift in the AgNCs.

Reported EOET values for noble metal nanoclusters vary widely—from 2.4 to 4.8 eV—depending strongly on synthesis method, cluster size, and surface functionalization.^21,32,80^ Our values are at the end of this range, consistent with the known trend that smaller metallic nanoclusters exhibit larger effective optical energy transitions.^32^ In fact, our PL results suggests that the excitation at about 300 nm is the main mechanism of light emission in the range of 410 to 420 nm, respectively, for silver and gold. This conclusion is supported by the observation that the only identified EOET derived from the Tauc's plot is near the 300 nm wavelength region.

Therefore, the similarity in emission energy and spectral position despite different surface chemistries suggests that the overall optical transition energy is governed predominantly by the electronic structure of the metallic cores, while surface oxidation states modulate the relative weight of the fast and slow decay channels and the fine spectral position, rather than the fundamental emission mechanism itself. It is worth noting that silver oxides behave more like semiconductors than metals: electrons become more localized, free-electron metallic behavior decreases, and wider forbidden energy regions appear. Thus a larger partial oxidation of Ag nanoclusters is consistent with the blue shift in absorption and the relative increase in effective optical bandgap. This can be observed by comparing the difference in the FWHM of the PLE of Ag and Au NCs with a maximum at 300 nm, where a difference equivalent to approximately 10 nm (0.2 eV) is exhibited.

## 4 Conclusions

Au and Ag NCs synthesized by PLAL at the argon–water interface exhibit nearly identical structural and optical properties—including photoluminescence, optical transitions, quantum yields, and lifetimes—within analogous ranges. The primary distinctions are attributable to the extent of oxidation and represent a possible impact on the equilibrium of light-emission decay channels, where a slight blue shift may be seen in the case of the AgNCs. AuNCs exhibit emission behavior that is primarily influenced by the rapid, core-related channel. Conversely, AgNCs exhibit a more balanced distribution of rapid and slow components in their decay.

In summary, these observations highlight the promise of Au and Ag NCs as model systems to enhance the fundamental understanding of light behavior in noble-metal nanostructures produced under a fully inert atmosphere (*i.e.*, argon) PLAL. Furthermore, they introduce novel inquiries into the impact of surface oxidation on electronic relaxation pathways of fully inorganic metallic nanoclusters. Moreover, they offer insight into the electronic structure of ultrasmall noble-metal clusters, highlighting the emergence of quantum-confined, molecule-like optical transitions also in nanostructures of the order of 3.0 nm, and offering a deeper understanding of the metal-to-cluster crossover at the nanoscale.

## Conflicts of interest

There are no conflicts to declare.

## Supplementary Material

NA-OLF-D6NA00468G-s001

## Data Availability

Data supporting this article are included in the main text and in the supplementary information (SI). Supplementary information: quantum yield measurements. See DOI: https://doi.org/10.1039/d6na00468g.
