# Supplementary material for: Structural and optical properties of ligand-free noble metal nanoclusters synthesized by ns-pulsed laser ablation at the argon–water interface
Source: Nanoscale Adv. 2026 Sep 15. Online ahead of print. doi: 10.1039/d6na00468g (PMC13607954; doi:10.1039/d6na00468g)
Supplement: Supplementary file 1 [file NA-OLF-D6NA00468G-s001.pdf]

Supplementary Information (SI) for Nanoscale Advances.
This journal is © The Royal Society of Chemistry

Supporting Information

Structural and optical properties of ligand-free noble metal nanoclusters synthesized by ns-pulsed laser ablation at the argon-water interface

Guilherme Conceição Concas[a,b*], Mariana Gisbert[a], Tahir[c], Natan Gonzaga dos Santos[a], Francesco Enrichi[b], Anna Safonova[b], Michele Cassetta[b], Sven Reichenberger[d], Theo Fromme[d], Alexandre Cuin[e], Ricardo Aucelio[f], Tatiana Dillenburg Saint'Pierre[f], Isabel Cristina dos Santos Carvalho[a], Wanessa Afonso de Andrade[a], Rajwali Khan[g], Zubair Ahmed[h], Sajid Farooq[i], Alexandre Mello[j], Nicola Daldosso[b], Tommaso Del Rosso[a*]

a) Department of Physics, Pontifical Catholic University of Rio de Janeiro/ Rua Marquês de São Vicente 225, 22451-900 Gávea, Rio de Janeiro, Brazil
b) Department of Engineering for Innovation Medicine, University of Verona, Strada le Grazie 15, 37134 Verona, Italy
c) Department of Physics, Pelotas Federal University, Rua dos Ipês - Capão do Leão, RS, 96050-500, Brazil
d) Department of Technical Chemistry I, University of Duisburg-Essen, Universitätsstraße 5, 45141 Essen, Germany
e) Departamento de Química, Instituto de Ciências Exatas, Universidade Federal de Juiz de Fora, Juiz de Fora, MG, 36036-900 Brazil
f) Department of Chemistry, Pontifical Catholic University of Rio de Janeiro, Rua Marquês de São Vicente 225, 22451-900 Gávea, Rio de Janeiro, Brazil
g) National Water and Energy Center, United Arab Emirates University, Al Ain, 15551, United Arab Emirates
h) School of Chemical Sciences, Universiti Sains Malaysia, Gelugor 11800, Pulau Pinang, Malaysia
i) College of Mathematical Medicine, Zhejiang Normal University, Jinhua, 321004, PR China
j) Laboratory of Surfaces and Nanostructures (LabSurf), Brazilian Center for Physics Research (CBPF), 22290-180 Rio de Janeiro, RJ, Brazil

Correspondence: tommaso@puc-rio.br; guilherme.conceicaoconcas@univr.it

S.1. Quantum Yield measurement

The photoluminescence quantum yield (QY) of the nanoclusters was determined using a comparative method. Rhodamine 101 in ethanol, which exhibits a QY of 100%, served as the reference standard [12]. Both the as-synthesized nanocluster samples and the reference were serially diluted to achieve several concentrations. Specifically, dilution ratios 1:2, 1:4, and a sample without dilution were prepared to maintain the absorbance below 0.1 for all

measurements. Each dilution was characterized by UV-Vis absorption spectroscopy and steady-state photoluminescence (PL) emission spectroscopy.

Representative emission spectra are presented in Figure S1. Excitation was performed at at 530 nm for the Rhodamine 101 reference.

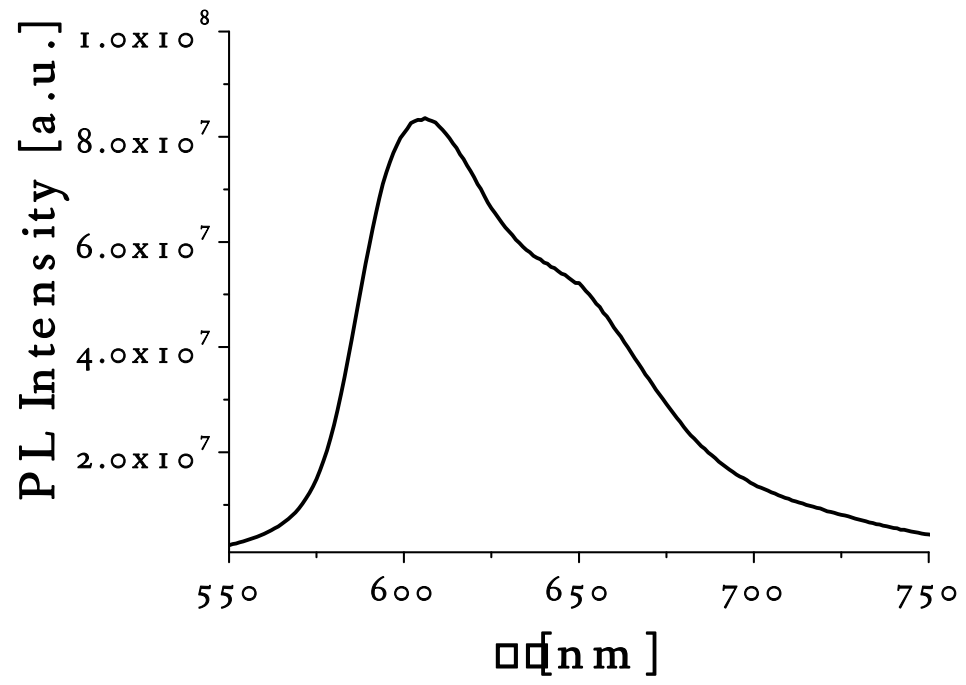

Figure S1. Representative photoluminescence emission spectrum of Rhodamine 101.

For QY calculation, the integrated PL emission intensities of both the reference and the synthesized samples (silver and gold nanoclusters) were plotted against their respective absorbance values at the corresponding excitation wavelengths (Figure S2(a,b)). The QY was then calculated using the following formula:

$$QY_s = \frac{S_S}{S_R} \cdot \left(\frac{n_s}{n_R}\right)^2 \cdot QY_R$$

where QY_R = 100% (Rhodamine 101 reference), S_S and S_R are the slopes of the integrated emission intensity versus absorbance plots for the sample and reference, respectively, and n_S and n_R are the refractive indices of the corresponding solvent media.

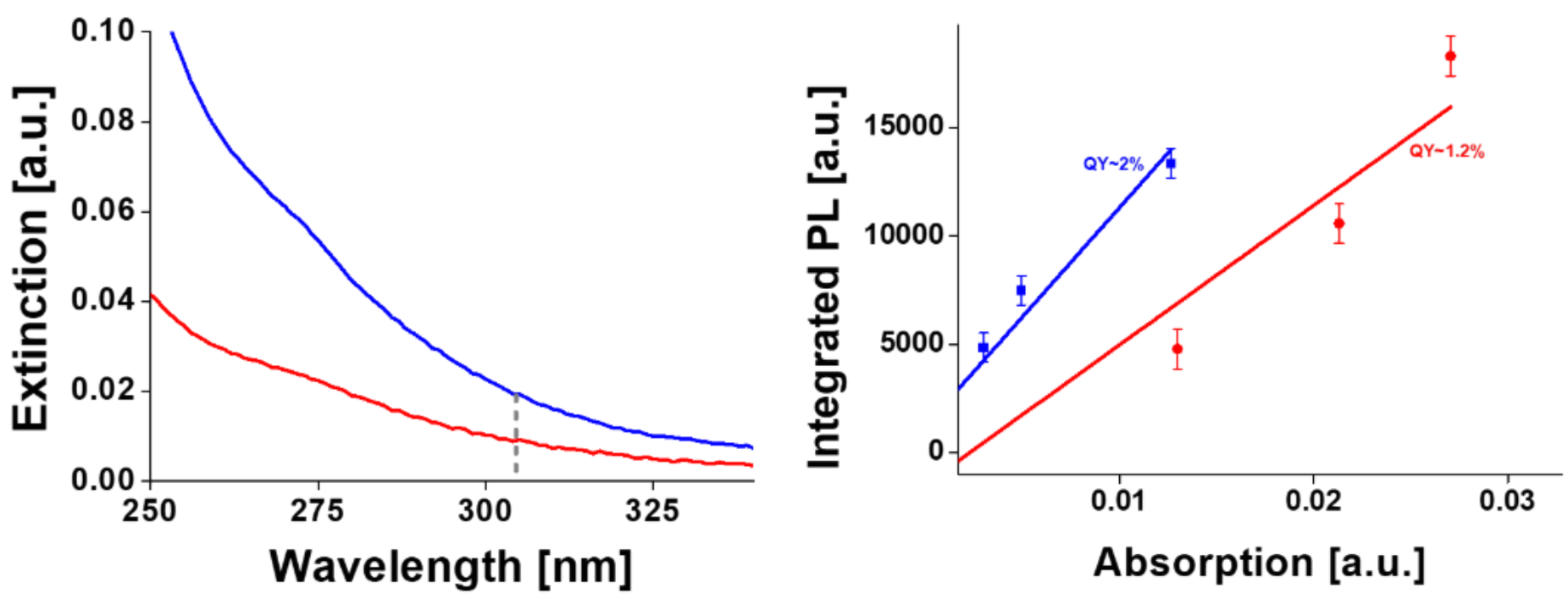

Figure S2. (a) Absorption (extinction) spectra of the photoluminescent Ag (blue) and Au (red) nanoclusters synthesized by PLAL at the argon/water interface after the centrifuge bleaching process. The vertical dashed gray line is located at 304 nm, corresponding to the wavelength used for the sample excitation. (b) Example of the curves used to calculate QY of the samples.
